# Strength Characteristics of Electrospun Coconut Fibre Reinforced Polylactic Acid: Experimental and Representative Volume Element (RVE) Prediction

**DOI:** 10.3390/ma15196676

**Published:** 2022-09-26

**Authors:** Olugbenga Ogunbiyi, Oluwashina Gbenebor, Smith Salifu, Samuel Olaleye, Tamba Jamiru, Rotimi Sadiku, Samson Adeosun

**Affiliations:** 1 Department of Mechanical and Mechatronics Engineering, Tshwane University of Technology, Pretoria 0001, South Africa; 2 Department of Metallurgical and Materials Engineering, University of Lagos, Akoka, Lagos 101017, Nigeria; 3 Center for Nanomechanics and Tribocorrosion, School of Mining, Metallurgy and Chemical Engineering, University of Johannesburg, Johannesburg 2006, South Africa; 4 Department of Mechanical Engineering, University of Lagos, Akoka, Lagos 101017, Nigeria; 5 Department of Chemical, Metallurgical and Materials Engineering, Tshwane University of Technology, Pretoria 0001, South Africa; 6 Department of Industrial Engineering, Durban University of Technology, Durban 4000, South Africa

**Keywords:** composite, electrospinning, mechanical properties, polylactide, RVE

## Abstract

Environmental conservation and waste control have informed and encouraged the use of biodegradable polymeric materials over synthetic non-biodegradable materials. It has been recognized that nano-sized biodegradable materials possess relatively good properties as compared to conventional micron-sized materials. However, the strength characteristics of these materials are inferior to fossil-based non-biodegradable materials. In this study, biodegradable polylactide (PLA), reinforced with treated coconut husk particulates (CCP) for improved mechanical properties, was fabricated using an electrospinning process and representative volume element (RVE) technique, and some of the obtained mechanical properties were compared. It was observed that the electrospun CCP-PLA nanofibre composites show improved mechanical properties, and some of these mechanical properties using both techniques compared favourably well. The electrospun fibres demonstrate superior properties, mostly at 4 wt.% reinforcement. Thus, achieving good mechanical properties utilising agro waste as reinforcement in PLA to manufacture nanocomposite materials by electrospinning method is feasible and provides insight into the development of biodegradable nanocomposite materials.

## 1. Introduction

Electrospun polylactic acid (PLA) fibres have recently been introduced in biomedical engineering to fabricate scaffolds in order to heal ruptured tissues [1,2]. The electrospinning method has gained wider acceptance in engineering due to improved properties often associated with the materials produced through the technique [3,4]. Polylactic acid is an important biodegradable polymer due to its low energy consumption and its being a non-toxic (to the environment) material [5,6]. Moreover, it satisfies the conditions for biocompatibility and biodegradability. However, the range of applications of PLA fibres is severely limited because of their low glass transition temperature [7,8], high brittleness, low toughness, slow crystallization rate, and low tensile elongation [9,10]. Additives, such as plasticizers, toughening agents, reinforcing fillers, and compatibilizers, have been found to widen the scope of applications of PLA by improving the necessary properties and crystallization rate [11,12,13]. On the other hand, many plants, crops, and pods from agricultural sources are considered important sources of viable natural fillers for polymeric composites to achieve better material and mechanical properties [14,15]. Owing to the increase in ecological safety practices and utilisation of renewable materials towards a greener society, the applications of natural fibres as bio-fillers in polymer composites by the relevant industries are increasing.

Recent studies have shown that natural fillers, as reinforcement in PLA, increase the crystallinity of electrospun PLA nanocomposite fibres, improving strength and decreasing fibre diameter, thus, significantly improving aspect ratio and surface area [16,17]. These nanofibres have qualities that make them useful as scaffolds in tissue engineering. The performance of these bio-fillers in polymer composites is influenced by factors such as fibres’ microfibrillar (orientation) angle, defects, structure, physical properties, chemical composition, cell dimensions, mechanical properties, and the interaction of fibres with the polymer matrix [15]. Several chemical modifications or pre-treatments of surfaces are constantly being explored and introduced in order to improve and enhance the adhesion or interfacial bonding between polymers and natural fibres [18,19]. Pre-treatments of natural fibres ensure that the fibre surface is clean and ready to modify the fibres’ surfaces; it decreases the rate of moisture absorption tendency and increases the external unevenness. The application of surface-treated fillers in the production of electrospun nanofibres is considered to be a productive technique for improving the properties of electrospun fibres in biomedical applications.

Furthermore, Finite Element (FE) micromechanical simulations, which are microstructure-based Representative Volume Elements (RVE), have been extensively implemented in the investigation of the mechanical performance of metal, ceramic, and polymer matrix composites [20,21]. The RVE is described as a volume significant enough to be statistically demonstrative of microstructural heterogeneities, especially for multiphase materials, and small enough to be deemed a volume element of continuum mechanics [22]. Therefore, RVE numerical simulation offers several advantages in investigating the effect of reinforcement with diverse particle size, dispersion, volume fraction and morphology, etc., especially on the response of composite materials to externally applied loads at the microscale level. The damage mechanism and microscale stress-train fields can also be evaluated during the loading process [21]. More importantly, 3D and 2D RVEs can be implemented in the FE simulation process. While the 2D simulation (showing plane stress or plane strain elements) displays attractive features compared to 3D and requires less time to calculate materials properties, it does not fully represent and capture the structure and stress–strain conditions of the materials. Thus, in the type of study under consideration in this paper, the 3D RVE is more suitable because it displays a high level of accuracy in the prediction of the mechanical properties of the developed composite as compared to 2D [23].

Similarly, the literature has established the benefit of using RVE in predicting the mechanical properties of ceramics/carbonaceous/fibre-reinforced metal/ceramic/polymer matrix composites [20,24]. However, limited studies that combine the experimental and computational investigation of agro-waste (coconut husk) reinforced PLA composite using electrospinning and RVE predicting methods are available in the open literature. Hence, this study presents the effect of coconut husk particulate reinforcement on the mechanical properties of reinforced PLA electrospun nanofibres and the FE micromechanical simulations through 3D RVEs. Mechanical and morphological properties are characterised by implementing a series of mechanical analyses and RVE responses. Concerning the simulation, a series of representative volume elements were produced automatically through different microstructural features, including the volume of reinforcement and the distribution of the reinforcement (throughout the composites), to assess their influence on the flow performance and damage progression in electrospun coconut husk/PLA under uniaxial tensile loading. Also, the modulus of elasticity, tensile energy-at-break, stress-at-break, and ultimate tensile strength of the electrospun scaffold composites featuring different microstructural characteristics was predicted from the RVE simulation and compared with the experimental results. Finally, water absorption analysis was conducted to assess the hydrophobic/hydrophilic characteristics of the electrospun nanocomposites scaffold produced.

## 2. Experimental and Representative Volume Element (RVE) Methodology

### 2.1. Materials

Industrial Polylactic acid, obtained from NatureWorks LLC (Beijing, China) supplier in China, was used in this study as the matrix. The coconut fibre was processed from coconut fruit received from the local market in Lagos (Lagos State), Nigeria. The solvent used in the electrospinning process was dichloromethane (DCM), manufactured by Shandong Jinhao Int’l Trade Co. Ltd., (Dongying, China) CAS number 75-09-2, 96% pure with a concentration of 14.9M.

### 2.2. Processing the Coconut Fibre

The coconut fibre was sun-dried for two weeks (average daily temperature of 31 ° C), after which the soft fibre was removed and cut into smaller pieces. Subsequently, it was dried for the second time and ground to pass through a screen size mesh of 10 mm in a mechanical crusher. The chemical composition of the coconuts’ husk particulates is presented in Table 1. It was subjected to a steam explosion at a temperature of 175 °C and pressure of 1 bar in an autoclave (SM280 E). Alkaline hydrolysis was conducted on the resulting fibre in a 2% solution of NaOH overnight, neutralized in acetic acid, and bleached with an 8% hydrogen peroxide solution. Further acid hydrolysis was undertaken with a mixture of 10% (*w*/*w*) nitric acid and 10% (*w*/*w*) chromic acid at a temperature of 60 °C for 15 min. Finally, the treated coconut fibres were pulverized to 75 µm.

### 2.3. PLA-Coconut Solution

The solutions of PLA were prepared in a glass bottle using the following technique: Glass bottle was first cleaned with tap water and then rinsed with DCM. Mixtures of PLA and treated coconut husk particulates (CCP) were dissolved in DCM at a constant composition of 12.50% (*w*/*v*). The solutions were left in sealed bottles for 24 h to dissolve the PLA and treated coconut husk particulates (CCP). The glass bottle was closed with an air-tight lid to prevent pollution and maintain concentration throughout the experiment. All solutions were prepared at room temperature 22 °C. This process was repeated for all the samples.

### 2.4. Electro Spinning of the CCP Reinforced Poly Lactide Nanocomposite Fibres

The solution samples of PLA and CCP in different weight percent (3–8%) were poured into a burette inclined at 30° to the horizontal surface. The flow rate was maintained at 0.01 mL/s at a constant voltage of 26 KV. The distance from the tip of the spinneret to the collector was kept constant at 24.5 cm. The electrospinning process was performed at 23 °C. As the charged polymer solution was ejected from the spinnerets, the solution jets evaporated to become nano fibres, which were collected onto an aluminium foil collector.

### 2.5. Representative Volume Element Methodology

In a bid to computationally predict the properties of coconut fibre reinforced PLA, representative volume element (RVE) software, Digimat, was used. During the composite development, the software assumes the following: (a) the matrix (PLA) and the inclusion (coconut fibre) are homogeneous, isotropic, and perfectly bonded to each other; (b) statistically, the distribution and orientation (random) of the coconut fibre with minute aspect ratio are homogeneous; (c) the applied load only induces elastic deformation [25]. It can be hypothesized, based on the stated assumption, that for any given inclusion (coconut fibre) with a fixed weight or volume fraction and small length to RVE size, the composite developed, and its properties, such as Poisson’s ratio and modulus of elasticity, are independent of the loading orientation [26].

**Figure 1:**
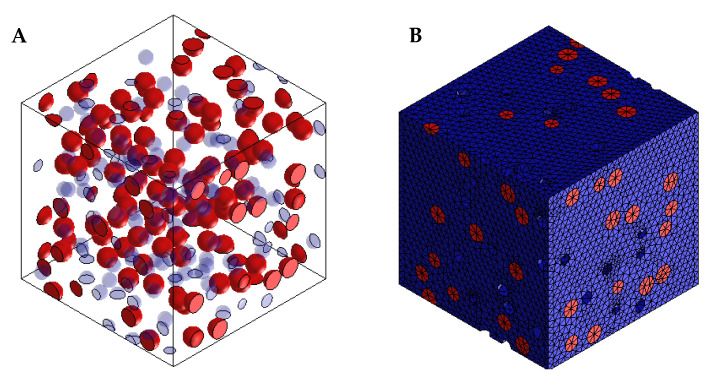
(**A**) Assembly and (**B**) mesh of model of PLA-coconut fibre reinforced composite.

Digimat-FE was selected from the arrays of packages in the Digimat software to develop the PLA-reinforced coconut fibre composites. The material properties of PLA and the coconut fibre used in this analysis are shown in Table 2. These properties are specified in the appropriate sections of the Digimat-FE software package. In the software, PLA was specified as the matrix, while coconut fibre was defined as the inclusion, and the appropriate void fractions, as computed from the experimental results, were incorporated in the simulation. Since the fabrication of the composite does not require heating of the mixture, mechanical analysis was selected. The shape of the coconut fibre (inclusion) used is ellipsoid, with an aspect ratio of 2.0, and the matrix-filler interface was assigned a perfect bonding. The developed model of the PLA-coconut fibre composite was meshed using a 0.05 mm mesh size, as shown in Figure 1.

**Figure 2:**
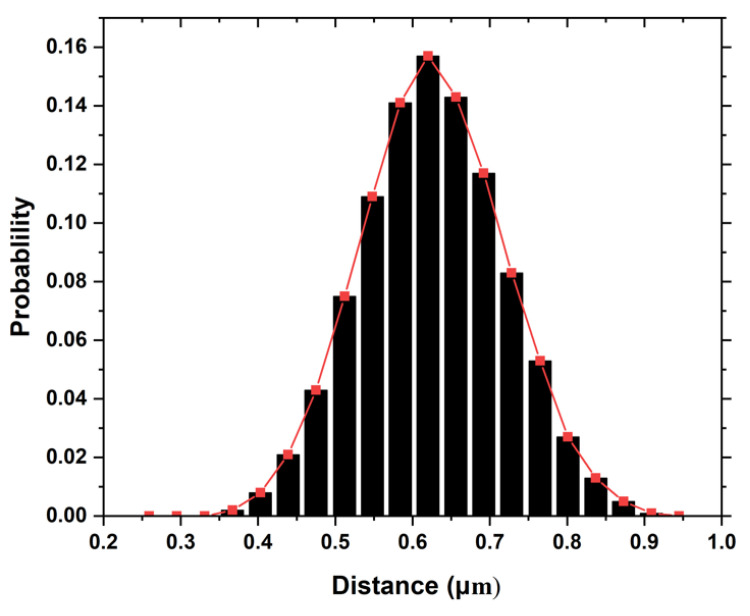
Normal distribution of the RVE for nearest neighbour distances of fibres in the composite with 3 wt.%.

In order to statistically quantify and characterize the distribution of fibre in the RVE, different statistical functions such as neighbouring distances, neighbour orientation, Voronoi polygon areas, radial function distribution, Ripley’s K function, etc. can be employed to describe the spatial distribution of fibres [27,28,29]. Studies have shown that inter-fibre distances greatly affect the developed stresses in the fibre/interface, and this affects the general behaviour of the developed composite [27,30]. In this study, the neighbour fibre distance was used by Digimat to quantify the fibre distribution of generated RVE. Generally, the nearest neighbour distance distribution is usually evaluated via the probability density function (PDF). Figure 2 shows the normal distribution of the neighbouring fibre distance across the developed polymer composite with 3 wt.% coconut fibre. The mean and standard of the normal distribution are 0.602 and 0.208, respectively.

To allow for homogeneity in the composite developed, the PLA-coconut fibre composite model was assigned a periodic boundary condition before running the analysis. The predicted bulk material properties of the developed composite are obtained in the Digimat software, and these properties are used in another finite element analysis software, Abaqus CAE, to predict the strength of the developed coconut fibre-reinforced composites.

### 2.6. Mechanical Test

Mechanical testing was performed using a double column Instron Universal tensile testing machine (grip jaw grabber), model 3369, located at the Center for Energy Research and Development (CERD), Obafemi Awolowo University, Ile-Ife, Osun State, Nigeria. The computerized mechanical testing machine had a load cell capacity of 50 KN with a continuous loading rate/strain rate of 5 mm per minute. Mechanical testing following the ASTM 1708D was performed three times for each composition (20 × 10 × 0.58 mm fibre specimens) [31], and the data set’s average was taken, representing the correct value measured. This is necessary to ensure data accuracy. For the simulated PLA-coconut fibre, the modulus of elasticity and density are obtained from the RVE software, Digimat.

**Figure 3:**
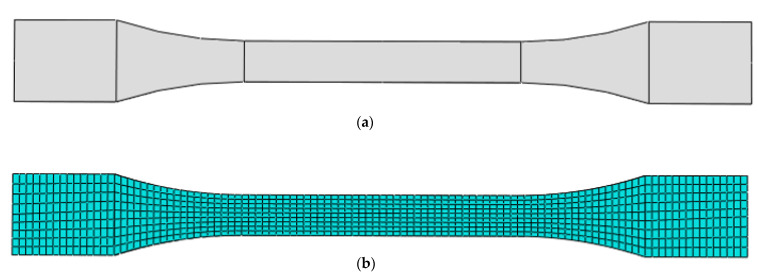
(**a**) part and (**b**) meshed model PLA-coconut fibre reinforced composite.

In contrast, other properties, such as the yield stress (stress at break), ultimate tensile strength, and strain energy, are obtained by exporting and using the bulk materials of the composite obtained from Digimat in another finite element analysis software, Abaqus. In the determination of these properties, the bulk material properties of the composite, as predicted in the Digimat analysis, were assigned to the developed tensile test model developed in Abaqus. Thereafter, the appropriate boundary conditions were applied in such a way that it mimics the real case scenario for conducting a tensile test. For the tensile test analysis, a dynamic explicit step was used in the tensile test [32], and the tensile model was assigned a 2.0 mm mesh size. Depicted in Figure 3 is the part and meshed model of the tensile specimen.

### 2.7. Morphological Examination

The morphology of the reinforced nanofibres was studied using an JEOL JSM-7600F Scanning Electron Microscope (SEM) (Freising, Germany), operated at an accelerating voltage of 15 kv. The samples were sputter-coated with a thin layer of silver to prevent the material from becoming charged by the electron beam during the analysis and improve conductivity. The micrographs of the treated CCP-PLA electrospun nanofibres at 0–8 wt.% were taken.

### 2.8. Water Absorption (Apparent Porosity)

A water absorption test was performed on all samples in order to determine the rate at which the CCP-PLA nano fibres absorb water. The samples were carefully dried in an oven at 35 °C for 1 h and weighed using the Unic Bloc Digital weighing balance (UW 1020H) with a tolerance of ±0.001 g, and then immersed in distilled water at 22 °C and a slightly elevated temperature of 70 °C. At room temperature, the samples were placed in different beakers containing a constant volume of distilled water for five days (120 h), with the water absorption rate measured at 24 h intervals. At 70 °C, the test was performed for two hours, with the absorption rate measured at 30 min intervals. The samples were periodically taken out of the water, wiped with tissue paper to remove surface water, and then weighed. The percentage of water absorption or weight gained (W%) was calculated using the expression in Equation (1).

W (%) = ( W₂ − W₁)/(W₁) × 100
(1)

where W_2_ is the weight gained after immersion and W_1_ is the initial weight before water immersion.

## 3. Results and Discussion

**Figure 4:**
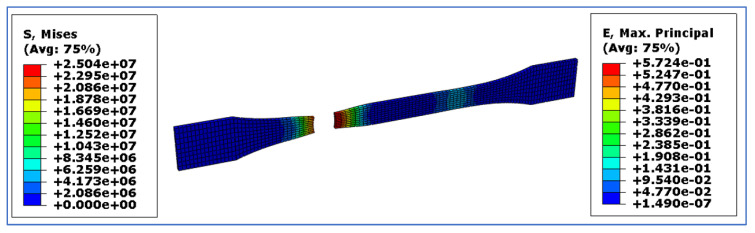
Von Mises stress and principal strain developed on the simulated tensile sample of the PLA-coconut fibre with 3 wt.% coconut.

The experimental and simulated results obtained from the RVE and finite element analysis software, Abaqus, are shown in this section. Figure 4 represents the stress and strain contour plot obtained for the tensile test conducted on the PLA-coconut fibre composite with 3 wt.% coconut fibre. Similar contour plot patterns were obtained with other percentage compositions of coconut fibre.

From the simulated tensile test of the composite with different weight percentages of coconut fibre, the different mechanical properties are compared with those obtained experimentally in subsequent subsections.

### 3.1. Ultimate Tensile Strength

**Figure 5:**
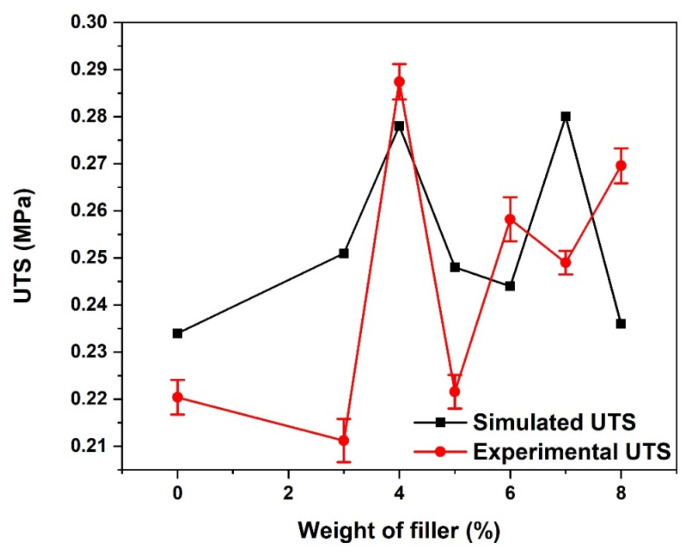
Ultimate tensile strength of CCP-PLA nanofibre.

As seen in Figure 5, both the simulated and experimental ultimate tensile strength (UTS) varies with an increase in the weight percentage of the reinforced composites. The reason for the irregular pattern observed in the UTS of the composite with an increase in reinforcement could be attributed to weak interfacial bonding and the presence of porosity [33,34]. At 4 wt.% coconut fibre addition, both the simulated and experimental composites gave the highest UTS, although there is a slight deviation in their values (0.278 and 0.287 MPa, respectively). With the further addition of coconut fibre, a decline in strength in both the simulated and experimental composites was observed. This is attributed to the large bead formations at these weight percentages (wt.%), as seen in the SEM image.

### 3.2. Tensile Energy-at-Break

**Figure 6:**
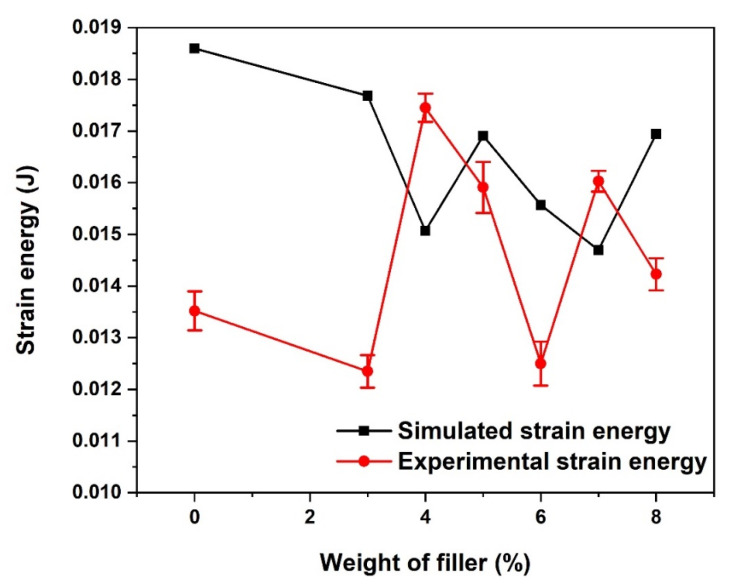
Energy-at-break of CCP-PLA nanofibre.

Just like the UTS, the strain energy at break was observed to vary randomly with an increase in the weight percentage of coconut fibre in the composites, as depicted in Figure 6. A significant difference was observed in the experimental and simulated composite strain energy with 0 wt.% coconut fibre reinforcement. Unlike the simulated strain energy, which appeared to be highest in the unreinforced composite, the experimental strain energy of the unreinforced composite is one of the smallest. The reason for the wide variation in the experimental and simulated strain energy could be attributed to the amount of void present in the electrospun composite used for the experimental analysis. The strain energy in the simulated composite with reinforcement was observed to decrease with an increase in the weight percentage of the fibre up to 4%, after which it started to increase gradually. A contrary pattern was obtained in the experimental strain energy, as the strain energy increased with an increase in the weight percentage of the fibre up to 4% before gradually decreasing with further addition.

### 3.3. Stress-at-Break

**Figure 7:**
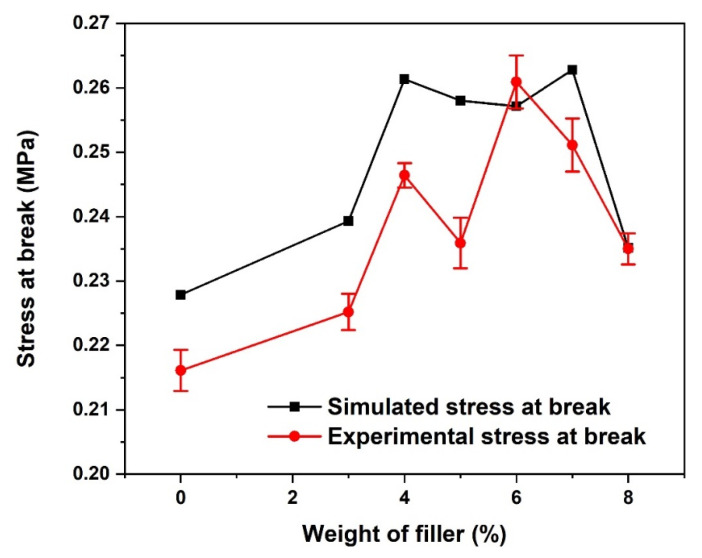
Stress at break of CCP-PLA nanofibre.

Depicted in Figure 7 are the fracture stress responses for both the simulated and experimental PLA-coconut fibre-reinforced composites. For both the simulated and experimental composites, the fracture stress response or stress at break increases with an increase in the weight percentage of the fibre up to 4 wt.% before gradually decreasing as more fibre is added up to 5 wt.% before increasing again and then decreasing. At 4 wt.% reinforcement, both the simulated and experimental composites have better fracture stress, of 0.261 and 0.246 MPa, respectively. However, the improved stress-at-break shown by the simulated composite compared to the experimental composite can be attributed to the controlled stronger interfacial bond between the reinforcement and the matrix polymer.

### 3.4. Young Modulus

**Figure 8:**
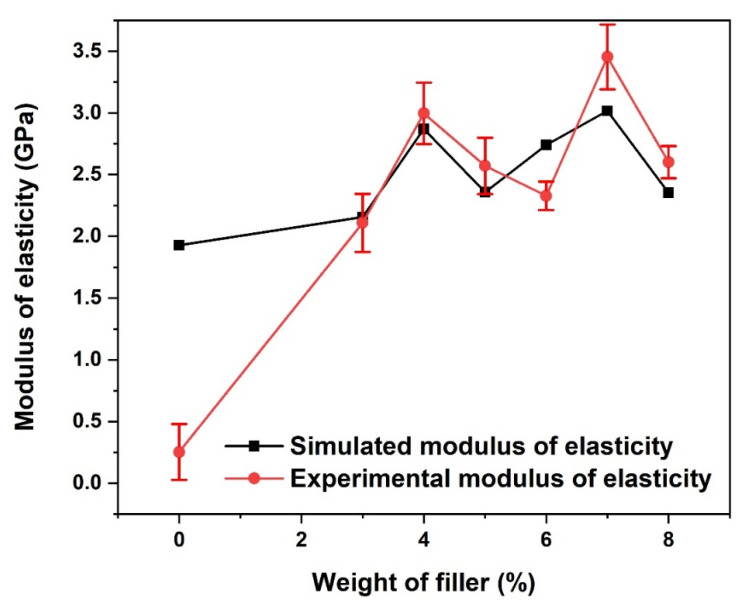
Young modulus of PLA-coconut fibre reinforced composite.

At almost all the weight percentages of the coconut fibre addition, the Young modulus of elasticity of the simulated and experimental PLA-coconut fibre-reinforced composites compared favourably well, as shown in Figure 8. In both cases, the highest Young modulus with 3.02 GPa for the simulated and 3.46 GPa for the experimental composite was obtained when 7 wt.% coconut fibre reinforcement was used. In general, the treatment of the fibres enhances the Young modulus because the acetylation of the fibres (i.e., the fibre treatment) improves the fibre–matrix interfacial bond [35]. The high Young modulus can also be linked to the high crystallinity and the good surface treatment that resulted, which led to the beneficial adhesion bonding properties experienced by the composites. Overall, the modulus of elasticity and strength of the electrospun nanofibre compared well with similar studies in the literature [31]. Thus, the treatment of coconut husk reinforcement improves crystallinity and adhesion with matrix PLA, yielding an improvement in the properties of the fabricated nanocomposite fibre compared to the untreated agro waste [36].

### 3.5. Water Absorption

**Figure 9:**
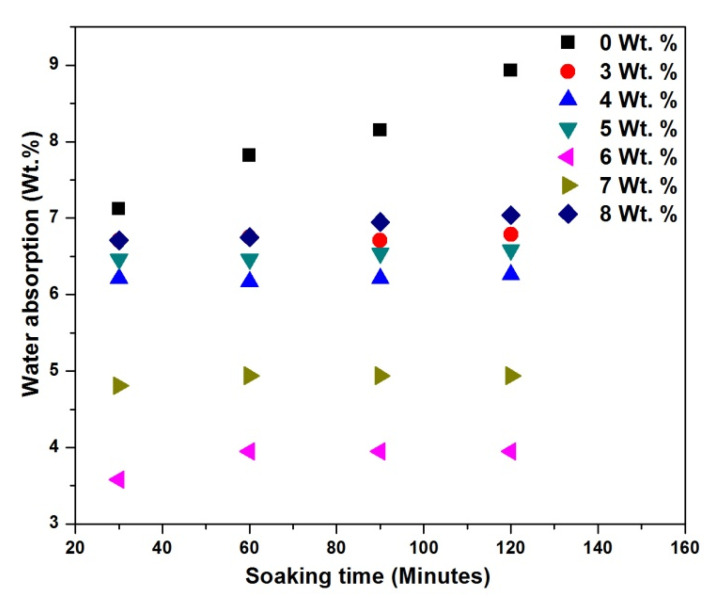
Water absorption of treated CCP-PLA nanofibre at 70 °C.

**Figure 10:**
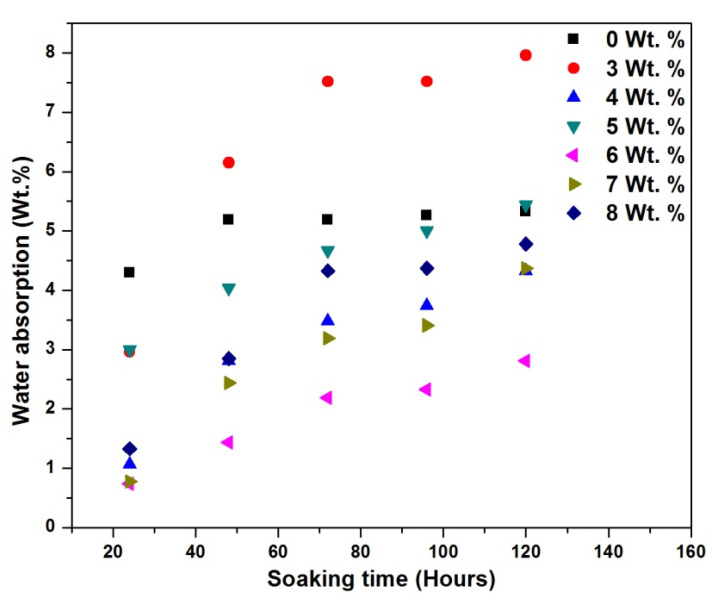
Water absorption of treated CCP-PLA nanofibre at 22 °C.

Figure 9 and Figure 10 show the water absorption rate as a function of time for different wt.% CCPs at 70 °C and 22 °C. Figure 9 shows that the water absorption rate appeared independent of wt.% CCP loading within 2 h in CCP-PLA nanofibre, unlike the responses in Figure 10, where there is a marked difference in variation in filler content in PLA. This result attests to the fact that the electrospinning process provides a relatively good dispersion of fillers and a good surface area-to-volume ratio. This helps the materials absorb water at a similar and comparable intensity [37]. This result also shows that even as the CCP is hydrophilic, electrospinning has helped reduce the water consumption rate, increase the bonding strength for the treated CCP-PLA nanofibres, and reduce the porosity level of the samples [38]. The improvement at 6 and 7 wt.% inclusions for the CCP-PLA nanofibres at 70 °C and 22 °C are attributed to the effect of the filler particles on the crystallization of the PLA matrix. They acted, essentially, as nucleating agents during the crystallization process. It is apparent that the filler led to increased crystallization of the polymer matrix and, therefore, decreased the matrix-free volume. Although, it is generally noted that a good dispersion of the filler increases the tendency to absorb water. The initial rate of water diffusion into the polymer matrix depends solely on the specific free volume of the polymer matrix; thus, the initial sharp increase in the rate of water absorption. Secondly, water absorption depends on the dispersion of the filler particles in the matrix; however, increasing agglomerations and bead formation may further increase the water absorption rate [39].

### 3.6. Morphological Structure

**Figure 11:**
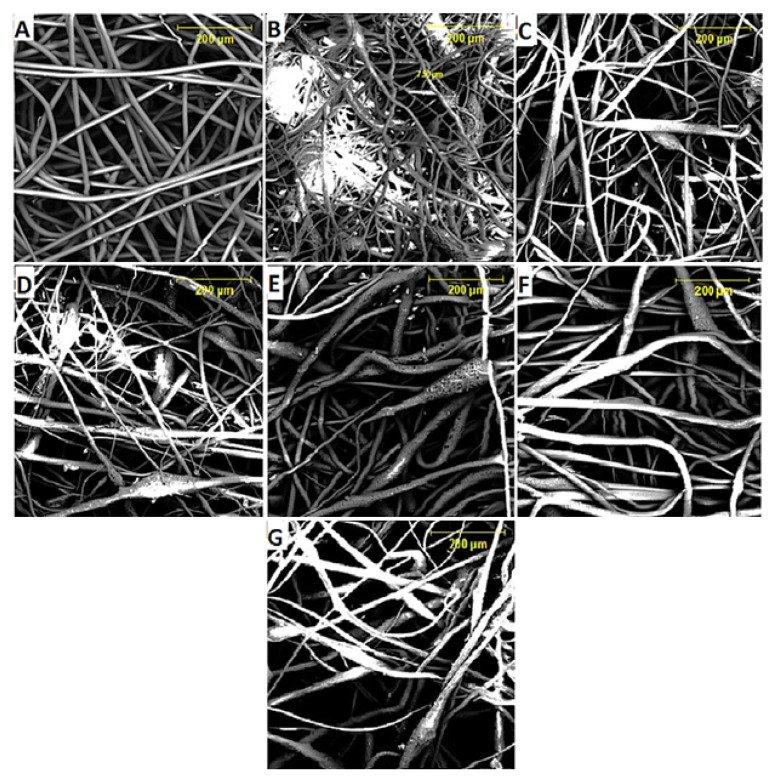
(**A**–**G**) SEM Morphologies of treated CCP-PLA nanofibres at different wt.% CCP: (**A**) 0, (**B**) 3, (**C**) 4, (**D**) 5, (**E**) 6, (**F**) 7, and (**G**) 8.

**Figure 12:**
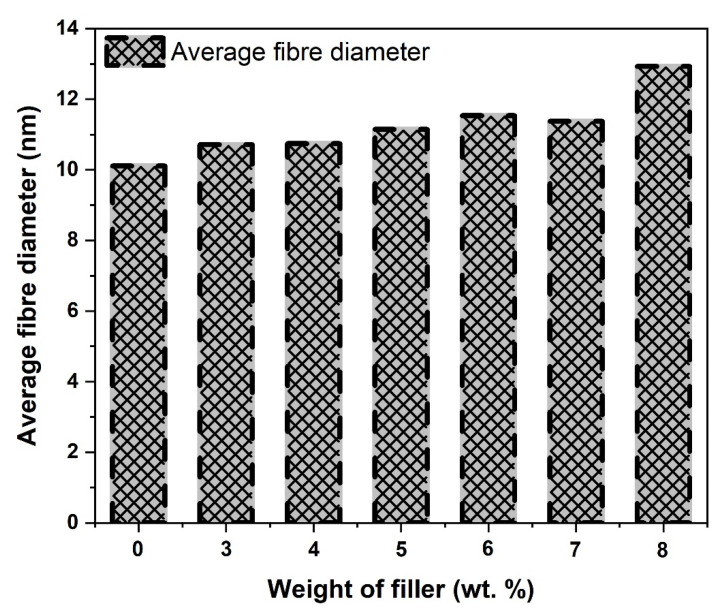
Average fibre diameter of CCP-PLA nanofibres.

Figure 11A–G presents the electrospun nanofibre scaffold morphology at different CCP reinforcement fractions from 0–8 wt.% and Figure 12 shows the average fibre diameter. Figure 11A represents 0 wt.% CCP reinforcement (pure PLA nanofibre), which displays dark, smooth, regular, cylindrical morphology with no beads and junctions. It indicates that the DCM solution evaporated quickly, and the nanofibre dried completely before reaching the collector. The electrospun nanofibres produced have smooth morphology with a wide range of diameters and an average fibre diameter of ~10.11 nm. The unreinforced nanofibre has a highest length of ~99.85 nm and a minimum length of ~12.61 nm. Pure PLA in solution has better electrical conductivity (free ions) when compared to its composites, which possess low electrical conductivity due to the CCP addition. Low conductivity reduces the charge in the electrospinning jet resulting in the formation of beads due to the solution not being fully stretched. This phenomenon occurred in the nanofibers formed, as seen in Figure 11B–G. The material conductivity characteristic is key to the formation of electrospun fibres; therefore, the virgin PLA possess high conductivity strength that allows the necessary increase in the stretching of the solution (dissolved PLA), and it yields fibres of smaller diameter [40]. Beads and junctions were observed in the treated CCP-PLA nanofibers (Figure 11B–G). This is attributed to the agglomeration of particles (hydrophilic nature of fibre) and random dispersion of CCP. There was an increase in electrospun fibre diameter with an increase in the concentration for treated CCP-PLA nanofibres (Figure 12) [41]. It was observed that the smaller diameter of CCP-PLA nanofibres at lower concentrations resulted from the solution being easily stretched during electrospinning. Interconnected pore networks were seen on the surface of the electrospun interwoven nanofibre mats. Also, the larger CCP-PLA nanofibre diameter at higher concentrations was attributed to the high viscosity of the solution that lowered the bending instability of the jet as the solution became resistant to stretching, occasioned by the electrical charge on the electrospinning [42]. Overall, the size of the CCP-PLA nanofibres was in the range of between 15.01 and 94.3 nm. It was shown that CCPs were well-loaded without any chemical and structural modifications into the PLA polymer matrix in order to produce the nanocomposite filler; hence, a well-dissolved and miscible solution resulted at room temperature after being left overnight to dissolve [43].

## 4. Conclusions

In this study, polylactide and coconut husk particle solution composites were successfully electrospun. Representative volume elements (RVE) and a finite element technique were employed to simulate the PLA-coconut fibre reinforced composites, and the mechanical properties obtained were compared with the electrospun scaffolds. The addition of coconut fibre resulted in a significant improvement in mechanical properties in both the electrospun and simulated composite, with 4 wt.% coconut fibre addition giving the best mechanical properties. The beads and junctions seen in the reinforced PLA micrograph were a result of the coconut fibre inclusion. Furthermore, reduced water absorption was also observed in composites with 6 and 7 wt.% treated fibres. Thus, filler treatment can significantly improve the adhesion between the filler and polymer matrix. Therefore, accomplishing good mechanical properties by employing agro waste as reinforcement in PLA to fabricate nanocomposite materials by electrospinning technique is achievable and provides understanding into the development of biodegradable nanocomposite materials.

## Figures and Tables

**Table 1:** Chemical composition of the coconut husk particulates.

Constituent	Lignin	Moisture	Pentosans	Ash	Cellulose	Solvent Extractives	Uronic Anhydrides
wt.%	29.4	8.0	27.7	0.6	26.8	4.2	3.5

**Table 2:** Material properties of PLA and coconut fibre used for RVE analysis.

Material	Density (kg/m^3^)	Modulus of Elasticity (GPa)	Poisson’s Ratio
PLA	1.250	3.420	0.3
Coconut Fibre	1.200	4.600	0.3

## Data Availability

Not applicable.
